# Chromosomal Conjugative and Mobilizable Elements in *Streptococcus suis*: Major Actors in the Spreading of Antimicrobial Resistance and Bacteriocin Synthesis Genes

**DOI:** 10.3390/pathogens9010022

**Published:** 2019-12-25

**Authors:** Virginie Libante, Yves Nombre, Charles Coluzzi, Johan Staub, Gérard Guédon, Marcelo Gottschalk, Sarah Teatero, Nahuel Fittipaldi, Nathalie Leblond-Bourget, Sophie Payot

**Affiliations:** 1 Université de Lorraine, Inra, DynAMic, F-54000 Nancy, France; 2 Groupe de Recherche sur les Maladies Infectieuses en Production Animale, Faculty of Veterinary Medicine, University of Montreal, Saint-Hyacinthe, QC J2S 2M2, Canada; 3 Public Health Ontario, Toronto Laboratory, Toronto, ON M5G 1M1, Canada; 4 Departments of Laboratory Medicine and Pathobiology, and of Cell and Systems Biology, University of Toronto, Toronto, ON M5S, Canada

**Keywords:** *Streptococcus suis*, integrative conjugative elements, integrative mobilizable elements, chromosomal excision, conjugative transfer, antimicrobial resistance, bacteriocin synthesis cluster

## Abstract

*Streptococcus suis* is a zoonotic pathogen suspected to be a reservoir of antimicrobial resistance (AMR) genes. The genomes of 214 strains of 27 serotypes were screened for AMR genes and chromosomal Mobile Genetic Elements (MGEs), in particular Integrative Conjugative Elements (ICEs) and Integrative Mobilizable Elements (IMEs). The functionality of two ICEs that host IMEs carrying AMR genes was investigated by excision tests and conjugation experiments. In silico search revealed 416 ICE-related and 457 IME-related elements. These MGEs exhibit an impressive diversity and plasticity with tandem accretions, integration of ICEs or IMEs inside ICEs and recombination between the elements. All of the detected 393 AMR genes are carried by MGEs. As previously described, ICEs are major vehicles of AMR genes in *S. suis*. Tn*5252*-related ICEs also appear to carry bacteriocin clusters. Furthermore, whereas the association of IME-AMR genes has never been described in *S. suis*, we found that most AMR genes are actually carried by IMEs. The autonomous transfer of an ICE to another bacterial species (*Streptococcus thermophilus*)—leading to the *cis*-mobilization of an IME carrying *tet*(O)—was obtained. These results show that besides ICEs, IMEs likely play a major role in the dissemination of AMR genes in *S. suis*.

## 1. Introduction

*Streptococcus suis*—a normal inhabitant of the upper respiratory tract of pigs—is an important cause of post-weaning infections in pigs and a zoonotic agent afflicting people in close contact with infected pigs or eating insufficiently cooked pork meat [1,2]. It is also a major agent of human meningitis in Asia [2]. In Western countries, cases are less frequent but can be severe and even fatal [3,4,5]. The most prevalent serotype causing human infection is serotype 2, although virulence differs among MultiLocus-Sequence-Typing (MLST) groups [2,5]. Serotype 14 strains are also frequently associated with human infections [2,5]. Moreover, a case of human infection due to serotype 9, which is increasingly reported in pig invasive disease worldwide, was reported in Thailand [6].

In the context of the rising threat of antimicrobial resistance [7,8] requiring a One Health approach, *S. suis* is receiving growing attention due to its extended drug resistance [9]. Numerous resistance determinants (in particular tetracycline, macrolide, aminoglycoside, and chloramphenicol resistance genes) have been described in this bacterial species, many of them being carried by Integrative Conjugative Elements (ICEs) [10,11,12,13,14,15,16]. ICEs are mobile genetic elements that excise from the chromosome and transfer autonomously by conjugation [17]. After cleavage at a site called *oriT*, DNA is addressed to a type IV secretion system by a relaxase, thanks to its interactions with a coupling protein [18]. The main families of ICEs carrying antimicrobial resistance (AMR) genes described in *S. suis* are Tn*916*, Tn*5252,* and Tn*1549* families [11,12,13,14,15,19,20]. Mosaic or composite elements resulting from tandem accretions of ICEs or internal insertions of ICEs within another ICE were reported [11,12,13,14,15,19,20]. Other extremely abundant but poorly documented Mobile Genetic Elements (MGEs) are Integrative and Mobilizable Elements (IMEs). IMEs were originally defined as excisable MGEs carrying their own *oriT* and encoding one or two mobilization proteins [21]. They can subvert the conjugation apparatus of unrelated conjugative elements to promote their own transfer [17,22,23]. According to their current definition, IMEs are not defective ICEs, i.e., elements deriving from these elements by deletion of parts of their conjugation modules. Furthermore, all known IMEs are unrelated or distantly related to ICEs [23]. So far, only one study examined the IME content of *S. suis* genomes (in 17 complete genomes), but cargo genes of these MGEs were not reported [22].

In this work, we made a comprehensive in silico search and analysis of ICEs and IMEs and extensive identification of AMR genes present in 214 *S. suis* draft genomes. We then analyzed whether these AMR genes are carried by MGEs and whether other genes of interest (bacteriocin synthesis clusters in particular) are found in MGEs. The ability to transfer of two ICEs that host IMEs carrying AMR genes was also investigated. A huge amount of ICEs and IMEs and derived elements were detected at various chromosomal sites (416 ICE-related and 457-IME-related elements). High diversity was observed in recombination but also in conjugation/mobilization modules. Besides ICEs, IMEs appear to be major vehicles of AMR genes. An IME carrying *tet*(O) was transferred from *S. suis* to *Streptococcus thermophilus* by *cis*-mobilization mediated by a Tn*5252*-related ICE.

## 2. Results

### 2.1. Prevalence and Diversity of ICEs and IMEs in S. suis Strains Belonging to Various Serotypes

Analysis of 214 genomes of *S. suis* strains belonging to 27 different serotypes revealed 232 ICEs with a full sequence, 49 ICEs with a partial sequence (due to sequencings gap(s) in the de novo assembled genome) and 135 elements deriving from ICEs (with truncated recombination and/or conjugation genes, called dICEs). IME-related elements appeared more prevalent than ICEs since 406 IMEs and 51 elements deriving from IMEs (with truncated integrase or relaxase gene, called dIMEs) were detected.

**Figure 1:**
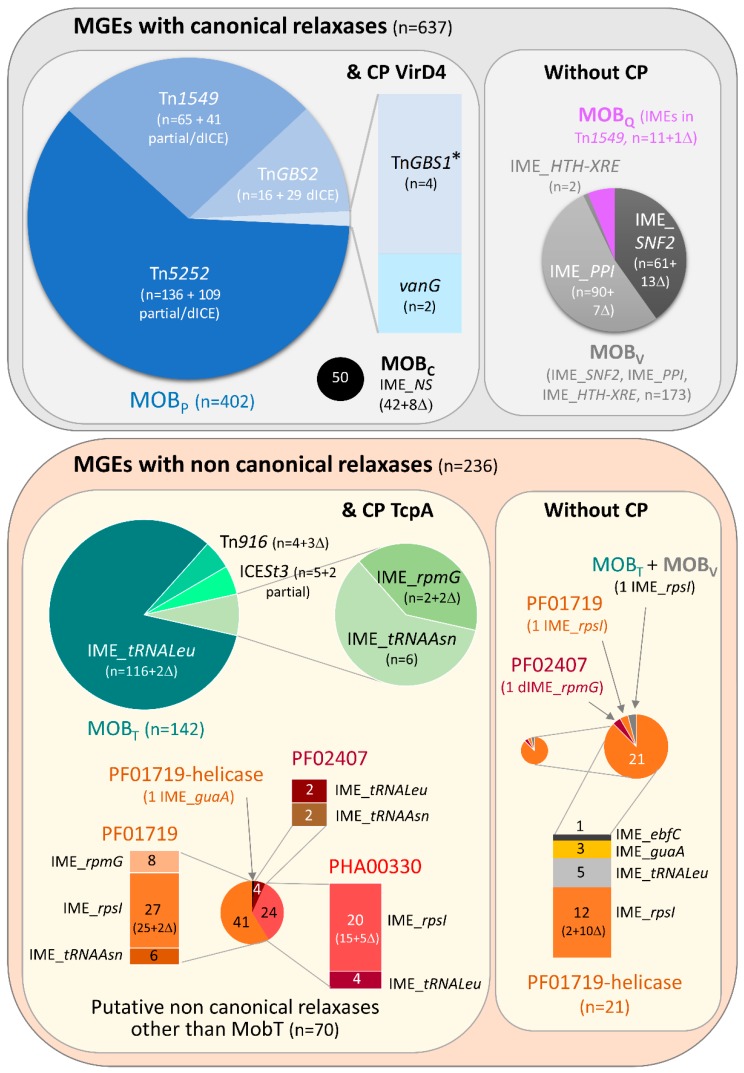
Description of the whole set of Integrative Conjugative Elements (ICEs), Integrative Mobilizable Elements (IMEs) and derived elements found in the 214 *S. suis* genomes analyzed. Elements were grouped according to the nature of their relaxase and the presence or absence of an associated coupling protein. * Tn*GBS1* is indicated in the elements with a MobP relaxase but it should be pointed out that its relaxase is only distantly related to MobP relaxases and does not possess the characteristic PF03432 domain of this family [23]. The sizes of the circles are at scale, except when zooming to provide further details. The integration sites of the IMEs are indicated in the element names themselves.

Most of the ICE-related elements found in *S. suis* genomes encoded a canonical relaxase of the MOB_P_ family (n = 402) associated with a coupling protein of the VirD4 family. By contrast, more diversity was observed for IMEs with canonical relaxases of the MOB_C_, MOB_V_ or MOB_Q_ families or putative non-canonical relaxases with various domains found in RCR initiators (PF02486 or MOB_T_, PF01719 associated or not with a helicase domain, PF02407, PHA00330 or MOB_T_ + MOB_V_). These relaxases were associated or not with a coupling protein (of the TcpA family except for those harboring a MOB_C_ relaxase that is associated with a VirD4 CP) (Figure 1 and Table S1).

Only one strain devoid of ICE and IME was identified. This strain did not possess any acquired resistance gene (Table S1).

#### 2.1.1. ICEs and Elements Deriving from ICEs

**Figure 2:**
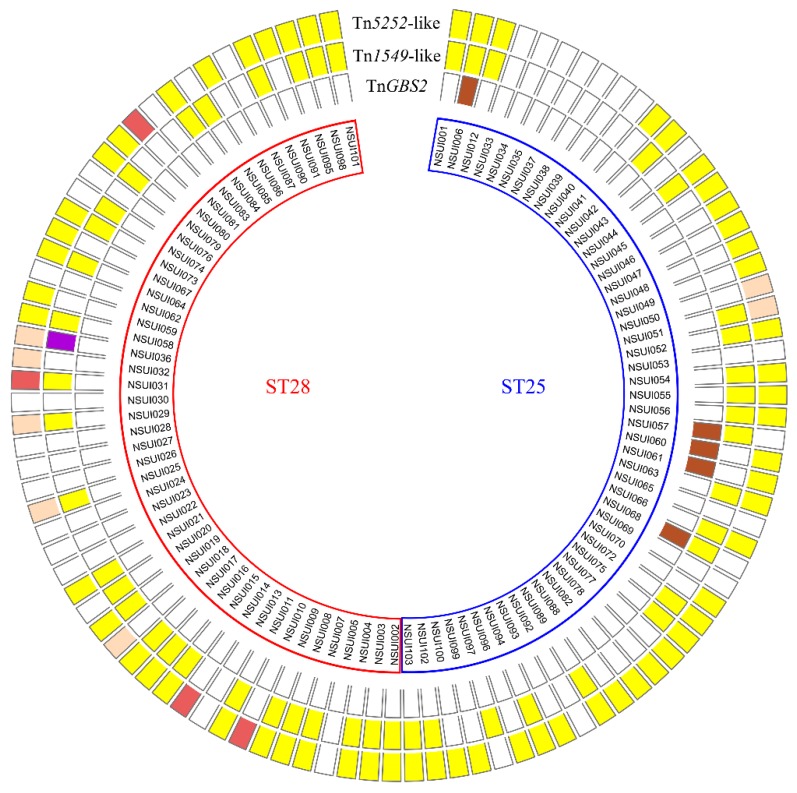
Integrative and conjugative elements detected in the genomes of *S. suis* strains belonging to serotype 2. Strains belonging to LultiLocus-Sequence-Typing (MLST) groups ST25 (n = 51, with a blue frame) and ST28 (n = 51, with a red frame) appear separately. Only ICEs with a full sequence are shown in the figure. Boxes located in front of a strain name are either empty (absence of ICE) or colored (presence of an ICE). Boxes in the outer circle, middle circle, and inner circle, correspond to ICEs belonging to Tn*5252*, Tn*154,9* and Tn*GBS2* families, respectively. The color of the box indicates the integration site of the element: yellow for integration into the 3′ end of *rplL*, salmon for integration into *rumA*, dark red for integration into *mutT*, purple for integration into the 5′ end of *rbgA*, dark brown for the lower specificity of Tn*GBS2*.

In strains of serotype 2 (belonging either to MLST groups ST25 or ST28), three families of ICEs (defined according to their conjugative module, i.e., >40% of identity for relaxase, CP and VirB4 protein [10]) were detected (Figure 2).

**Figure 3:**
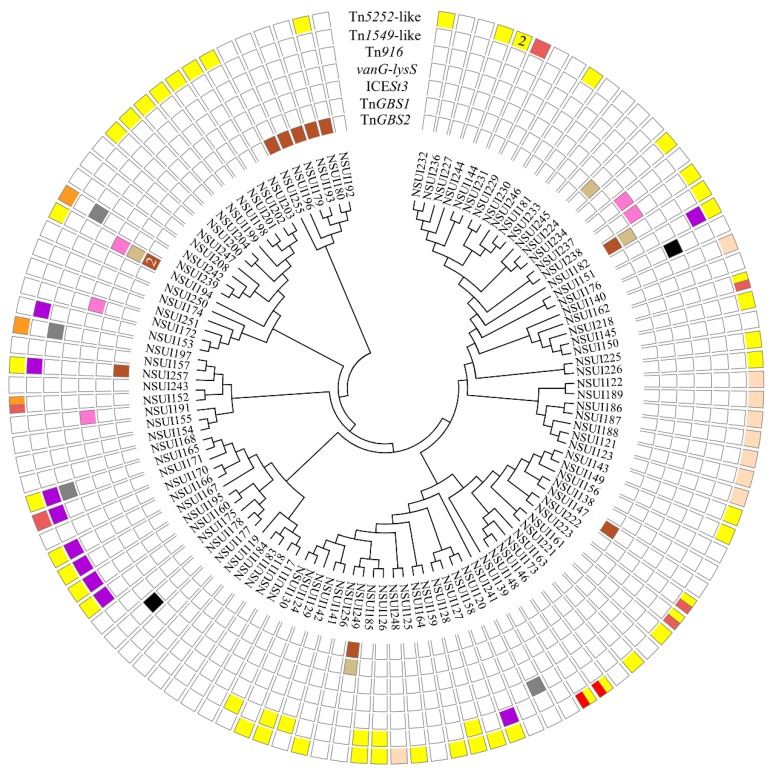
Integrative and conjugative elements detected in the genomes of *S. suis* strains belonging to other serotypes than serotype 2. Strains were grouped according to their MLST alleles. The cladogram was generated by maximum likelihood method on concatenated sequences of the *aroA*, *cpn60*, *dpr*, *gki*, *mutS*, *recA,* and *thrA* genes. Each family of ICEs is indicated on a specific circle. The color of the box indicates the integration site of the element. For elements of the Tn*5252* family (first outer circle): yellow is used for integration into *rplL*, salmon for integration inside *rumA*, dark red for integration inside *mutT*, red for integration in the ADP ribose pyrophosphatase gene and orange for integration in the luciferase monooxygenase gene. For elements of the Tn*1549* family (second outer circle), yellow is used for integration into the 3′ end of *rplL* and purple for integration into the 5′ end of *rbgA*. On the third outside circle, elements of the Tn*916* family are indicated in dark grey. Elements of the *vanG* family integrated into the 3′ end of *lysS* are indicated in black on the 4th circle (middle circle) and elements of the ICE*St3* family integrated into the 3′ end of tRNALys on the 5th circle (pink color). Elements of the Tn*GBS1* and Tn*GBS2* families are indicated on the 6th and 7th inner circles respectively (light and dark brown colors). When several elements of the same family are integrated in different sites in the same strain, the corresponding box is bicolor (for example 2 elements of Tn*5252* family in NSUI218, one integrated into *rplL* and one integrated inside *mutT*) whereas if they are inserted in the same site the number of elements is indicated (for example 2 elements of Tn*5252* family in NSUI144 both integrated into the *rplL* gene).

In the other serotypes examined, seven families of ICEs were detected: Tn*5252*, Tn*1549,* and Tn*GBS2* families and four additional ones: Tn*916*, *vanG*, ICE*St3*, and Tn*GBS1* families (Figure 3).

In total, considering all the genomes studied, Tn*5252*-related ICEs were the most prevalent ones (n = 136 ICEs with full sequence). They were found integrated either into the 3′ end of *rplL* (gene encoding the ribosomal protein L7/L12), inside *rumA* (gene encoding an RNA methyltransferase), inside *mutT* (gene encoding a NTP pyrophosphohydrolase), inside a gene encoding a luciferase-like monooxygenase or inside a gene encoding an ADP ribose pyrophosphatase (Figure 2). Partial ICEs or elements deriving from ICEs by deletion were also detected at the 5′ end of *rbgA* (gene encoding a ribosomal biogenesis GTPase) (Table S1).

Most of the Tn*1549*-related elements (49 out of 65 elements found in total) were integrated in tandem with a Tn*5252*-related element (into *rplL*). We also found that two ICEs (or elements deriving from ICEs) of the same family (Tn*5252* or Tn*1549* families) can co-exist in the same strain.

All the detected 16 Tn*GBS2*-related ICEs were integrated into intergenic regions. None were found in the *rplL* integration site.

The four ICEs of the Tn*916* family detected in this work include two Tn*916* (with one integrated into an ICE of the Tn*5252* family) and two ICEs with a conjugation module distantly related to Tn*916* (integrated inside a tRNAArg gene).

ICEs of the ICE*St3*, Tn*GBS1,* and *vanG* families were found in 5, 4, and 2 genomes, respectively.

#### 2.1.2. IMEs and Elements Deriving from IMEs

IMEs were detected in a large panel of chromosomal integration sites (13 different integration specificities as reported in Table S1). Half of these integrations (n = 6) relied on tyrosine integrases that mostly target genes encoding tRNAs (tRNALeu or tRNAAsn) or ribosomal proteins (mostly in *rpsI*, several in *rpmG* but none in *rplL*). Five cases of IME-IME accretions were detected (in tRNALeu and *rpsI* sites). The second half of integrations (n = 7) was mediated by serine recombinases. Interestingly, some of them target conserved genes located on ICEs including two genes of the Tn*5252* family: the SNF2 gene encoding a putative adenine-specific DNA methylase with a COG4646 domain and a helicase domain (n = 61) and a gene encoding a putative peptidylprolyl isomerase (PPI) (n = 90).

**Figure 4:**
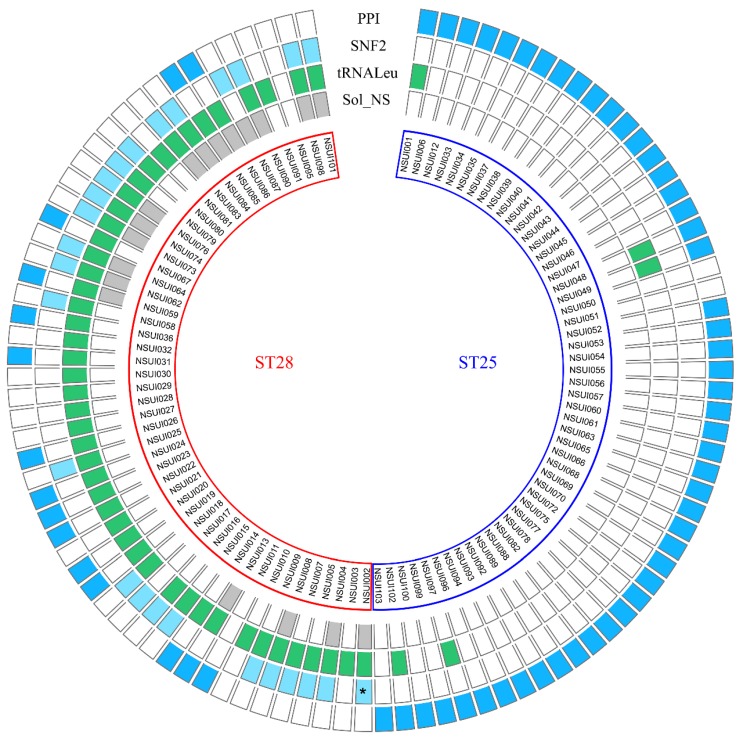
Integrative and mobilizable elements detected in the genomes of *S. suis* strains of serotype 2. Boxes located on the same line as a strain name are either empty (absence of IME) or colored (presence of an IME). Strains belonging to MLST groups ST25 (n = 51) and ST28 (n = 51) are grouped in the figure (blue and red frames, respectively). From the outer to the inner: IMEs integrated in the peptidylprolyl isomerase gene (in dark blue on the first outside circle); IMEs integrated into SNF2 gene (in light blue on the second circle); IMEs integrated into a tRNALeu gene (in green on the third circle); IMEs integrated into intergenic regions (in light grey on the inner circle). One element resulting from recombination between an IME_*SNF2* and an IME_*PPI* (called “hybrid” in Table S1) is indicated by a star.

For strains of serotype 2, four different integration specificities were detected (Figure 4). All strains belonging to MLST group ST25 (except two) carry an IME integrated inside the PPI gene whereas most of the ST28 strains carry an IME integrated inside the SNF2 gene. ST28 strains also frequently harbor IMEs integrated into a tRNALeu gene or IMEs with a low specificity of integration.

**Figure 5:**
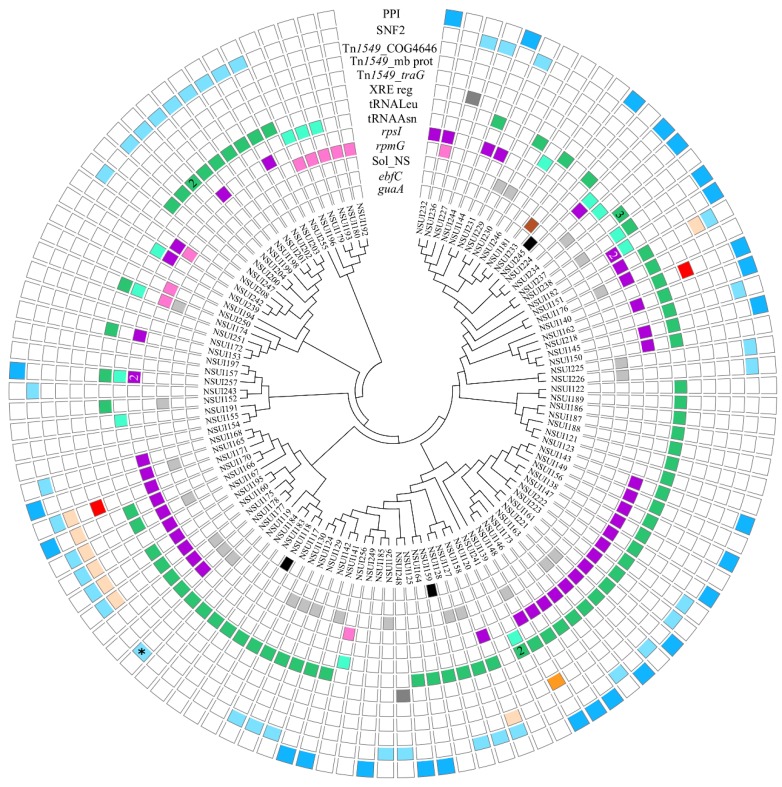
Integrative and mobilizable elements detected in the genomes of *S. suis* strains of serotypes other than 2. Boxes located on the same line as a strain name are either empty (absence of IME) or colored (presence of an IME). Strains were grouped according to their MLST alleles (see material and method section for the method used to generate the cladogram that appears in the middle of the figure). The first two outer circles indicate IMEs integrated into the peptidylprolyl isomerase gene (in dark blue) and IMEs integrated into the SNF2 gene (in light blue) (IMEs integrated into ICEs belonging to the Tn*5252* family). The third, 4th and 5th circles show IMEs integrated into ICEs of the Tn*1549* family (from outside to inside: in a gene encoding a protein with a COG4646 domain shown with a salmon color, a membrane protein shown in orange and in *traG* shown in red). The next circle indicates the presence of IMEs integrated into an XRE regulator gene (dark grey color). Middle circles show IMEs integrated into the 3′ end of tRNA genes; in green for integration into tRNALeu gene (7th circle) and in light green for tRNAAsn gene (8th circle). On the following circles (still from outside to inside): IMEs integrated into *rpsI* (in purple on the 9th circle) and into *rpmG* (in pink on the 10th circle). Inner circles indicate IMEs with a low specificity of integration (in light grey on the 11th circle), in *ebfC* gene (in dark brown on the 12th circle) and in *guaA* (in black on the 13th last inner circle). One element resulting from recombination between an IME_*SNF2* and an IME_*PPI* (called “hybrid” in Table S1) is indicated by a star. When several elements are present at the same integration site, the number of elements is indicated in the box (for example, 3 IME_*tRNALeu* in strain NSUI237).

In the other serotypes examined, a large diversity of IMEs was detected. In addition to the four integration specificities already detected in strains of serotype 2, nine additional ones were found (Figure 5). IMEs that target the SNF2 and PPI genes were detected in ICEs integrated into either *rplL*, inside *rumA* or inside *mutT*. IMEs integrated inside genes of Tn*1549*-related ICEs were also detected: (i) inside a gene that encodes a protein distantly related to SNF2 of Tn*5252* (indicated as Tn*1549*_COG4646); (ii) inside a gene encoding a membrane protein (Tn*1549*_mb prot), and (iii) inside a gene encoding the coupling protein of the ICE (Tn*1549*_*traG*). IMEs encoding a serine integrase were also detected inside a gene encoding an HTH_XRE regulator and at various intergenic sites for elements encoding related serine integrases (Table S1, Figure 5).

**Figure 6:**
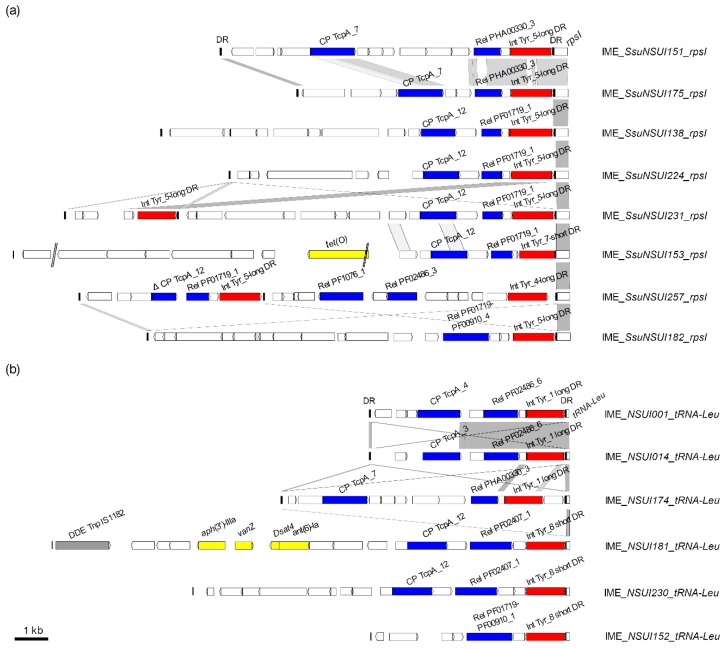
Comparison of integrative and mobilizable elements (IMEs) integrated (**a**) into *rpsI* and (**b**) into a tRNALeu gene in *S. suis*. IMEs are named according to their host strains and integration sites. Direct repeats are indicated as black vertical rectangles. Coding DNA sequences (CDSs) appear as arrows (truncated genes are indicated by deltas). The integrase genes appear in red. The relaxase and coupling protein genes appear in blue. The different integration genes (*rpsI* and tRNALeu gene) targeted by the integrase are indicated in the figure and are part of the IME name. Genes encoding proteins with putative function inferred from in silico analysis are indicated (in yellow for antimicrobial resistance genes and in gray for transposase genes). Nucleic acid sequence identity between sequences is indicated in light gray when >80% of identity and in dark gray when higher than 90% of identity. Gaps in the assembly are indicated by a double slash.

It is difficult to define families for IMEs since a relaxase of a given family (defined according to phylogenetic analysis and 40% sequence identity clustering [22]) can be associated with different families of coupling proteins (for example, relaxase with a PF02486_6 domain can be associated with two different CPs in *S. suis* IMEs, Figure S1). In the same way, a coupling protein of a given family (for example, a TcpA_12 coupling protein) can be associated with different families of relaxases (2 different relaxases for the example of TcpA_12, Figure S1). In total, twenty-two different combinations of integrase-relaxase-(coupling protein) were observed for IMEs (Figure S1). The highest diversity was observed for IMEs integrated into *rpsI* and into a tRNALeu gene (Table S1, Figure 6).

### 2.2. Antimicrobial Resistance Genes Carried by Chromosomal MGEs in S. suis

All acquired AMR genes (n = 393) detected in the 214 genomes of *S. suis* were located on MGEs (Table S1). For strains of serotype 2 and MLST group ST25, the detected resistance genes support previously described phenotypic antimicrobial susceptibility determinations [11]. In addition, to select strains for downstream analysis (see below), the susceptibility to chloramphenicol, kanamycin, tetracycline, lincomycin, and erythromycin of 14 strains of serotype 2/MLST group ST28 was evaluated in this study. In all cases, resistance genes matching the observed resistance phenotype were detected by in silico analysis, thus supporting the notion that these genes are fully functional and expressed by the corresponding *S. suis* strains.

#### 2.2.1. ICEs carrying AMR Genes in *S. suis*

A total of 55 AMR genes were found on ICEs (excluding ICEs hosting IMEs carrying AMR genes).

**Figure 7:**
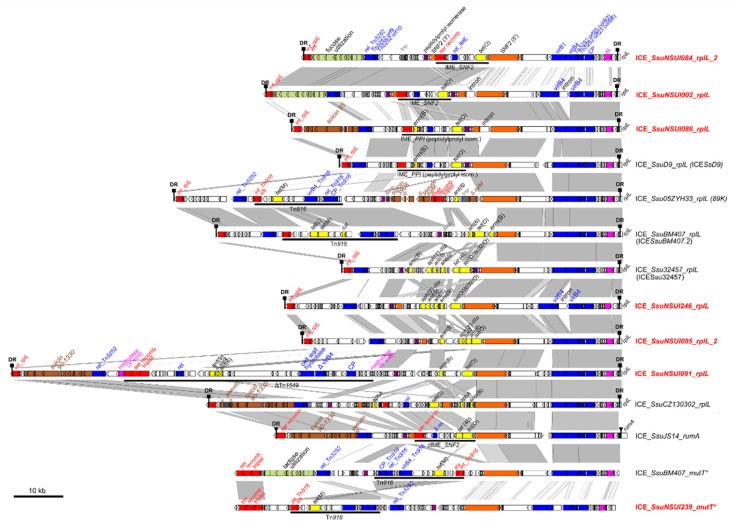
Comparison of the integrative and conjugative elements (ICEs) belonging to the Tn*5252* family and carrying AMR genes found in *S. suis*. ICEs are named according to their host strains and integration sites. When another name has already been published for the element, it is indicated in brackets. ICEs identified in this work are indicated in red. For more clarity, elements in accretion with ICEs of the Tn*5252* family are not shown in the figure. ICEs or IMEs integrated inside ICEs are indicated as black lines with an indication of the name of the element. Nucleic acid sequence identity higher than 80% between two sequences is indicated in light gray and that higher than 90% in dark gray. Direct repeats (DR) delimiting ICEs are shown as squares or triangles depending on the integration gene (*rplL*, *rumA* or *mutT* *). Coding DNA sequences (CDSs) appear as arrows (truncated genes are indicated by deltas). Modules of recombination (integrase [*int*], excisionase [*xis*] and serine recombinase genes [ser recomb]) and conjugation appear in red and blue, respectively. The different integration sites (*rplL*, *rumA*, *mutT* *) targeted by the integrase are indicated in the figure and are part of the ICE name. Genes encoding proteins with putative function inferred from in silico analysis are indicated in yellow for antimicrobial resistance genes, in orange for SNF2, in purple for the peptidylprolyl isomerase gene, in green for sugar metabolism cluster, in pink for methylase genes, in gray for transposase genes, and in brown for bacteriocin synthesis clusters.

Most of the ICEs carrying AMR genes belong to the Tn*5252* family (see Figure 7 for representative ICEs of this family).

**Figure 8:**
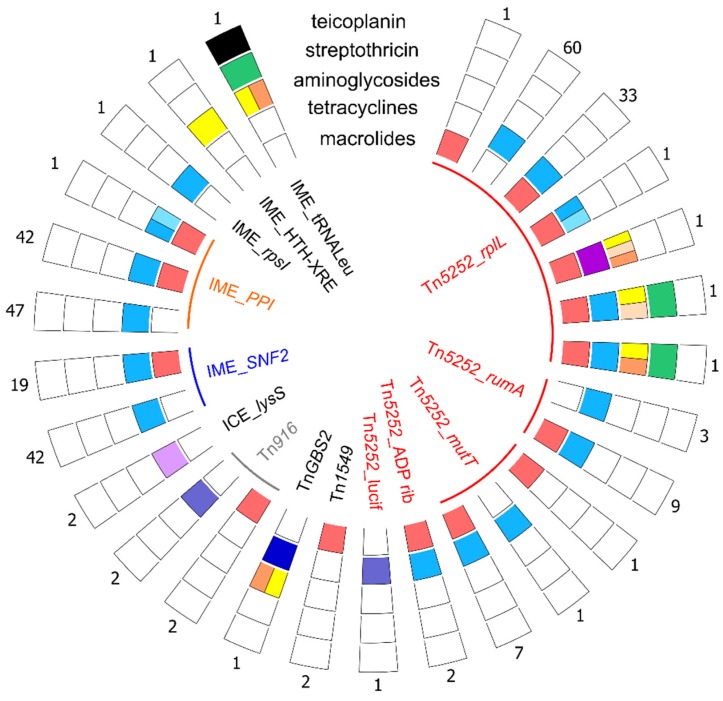
ICEs and IMEs carrying AMR genes in the 214 genomes of *S. suis* analyzed. Each panel of boxes represents the combination of AMR genes present on the corresponding ICE or IME indicated in the inner circle. ICEs are grouped by families (with an indication of the integration site for ICEs of the Tn*5252* family) and IMEs by integration sites. Each outer circle corresponds to resistance genes against a family of antibiotics: teicoplanin, streptothricin, aminoglycosides, tetracyclines and macrolides (from outside to inside). Different colors are used for the boxes to distinguish the AMR genes: black for *vanZ*, green for *sat4*, yellow for *ant*(6)-Ia, salmon for *ant*(9)-Ia, orange for *aph*(3′)-IIIa, light blue for *tet*(40), blue for *tet*(O), darker blue for *tet*(M) (on Tn*916*) and *tet*(L) (on Tn*GBS2*), light purple for *tet*(W) and deep purple for *tet*(O/W/32/O) and red for *erm*(B). The number of corresponding elements is indicated at the extremity of each panel of boxes. ICEs of the Tn*5252* family are all counted even if the AMR genes are carried by IMEs integrated inside a conserved gene of the ICEs.

AMR genes were also found on four other ICE families (Figure 8). The first one is the *vanG* family with elements integrated into a *lysS* gene (with a *tet*(W) gene). The second one is the Tn*GBS2* family (one ICE with *tet*(L)-*ant*(6)-Ia-*aph*(3′)-IIIa genes). Interestingly, a thorough analysis of the Tn*GBS2*-related ICE indicated that the cluster of AMR genes could have been brought by the integration of an IME inside the ICE. Indeed, a gene encoding a serine recombinase was detected as well as a gene encoding a relaxase with a PF01076 domain (Mob_V_ family). The third family is the Tn*916* family with two Tn*916* and two ICEs (carrying an *erm*(B) gene) with a conjugation module distantly related to the one of Tn*916* that are integrated into a tRNAArg gene. These latter are almost identical to Tn*6194* previously characterized in *Clostridioides difficile* CII7 [24]. The fourth family is the Tn*1549* family with two ICEs (carrying *erm*(B)) found in *rbgA* and a dICE (with *ant*(6)-Ia-*ant*(9)-Ia-Δ*sat4* genes) integrated in a newly reported integration site i.e., a gene encoding a methylase located on a Tn*5252*-related ICE (Figure 8 and Table S1).

#### 2.2.2. IMEs Carrying AMR Genes in *S. suis*

More than half of the detected AMR genes (n = 221) were found on putative IMEs.

The mobilization modules of these IMEs carrying AMR genes include unrelated relaxases belonging to three families (Table S1).

Most of them carry *tet*(O) (that confers resistance to tetracycline) (n = 89) or *tet*(O) and *erm*(B) (that confers resistance to macrolides-lincosamides-streptogramin B) (n = 62) (Figure 8) and are themselves carried by ICEs of the Tn*5252* family. They are integrated into SNF2 (for example, in ICE_*SsuNSUI002_rplL*, Figure 7) or into PPI (for example, in ICE_*SsuNSUI086_rplL* and the previously described ICE_*SsD9* [14], Figure 7).

IMEs carrying AMR genes were also detected in three additional integration sites in *S. suis* genomes: one in the *rpsI* gene (carrying a *tet*(O) gene), one in a gene encoding a putative HTH-XRE regulator (with *ant*(6)-Ia), and one in a tRNALeu gene. This latter carries four different AMR genes: *aph*(3’)-IIIa-*vanZ*-*sat4*-*ant*(6)-Ia (Figure 8) conferring resistance to kanamycin, teicoplanin (low-resistance), streptothricin, and streptomycin, respectively. The encoded VanZ protein displays only 38% of identity with the one previously described in *S. suis* GZ0565 [25]. However, interestingly, it shows 100% of identity with the one encoded by an IME integrated inside a Tn*1549*-related ICE—itself integrated with a Tn*5252*-related ICE—in *Streptococcus lutetiensis* (ICE*Slu*van) [17,26]. These two IMEs carrying *vanZ* differ by their integrase and mobilization proteins (different relaxase and presence of a coupling protein only for the IME integrated into a tRNALeu gene).

#### 2.2.3. Other MGEs Carrying AMR Genes in *S. suis*

AMR genes were also found on prophages (n = 33, Table S1), most of them being integrated in rumA.

AMR genes were also located on genomic islands (GI) which correspond either to partial ICEs or elements deriving from ICEs (n = 40) or to elements for which no relaxase has been detected and were not categorized as IMEs in this work (n = 45, called GI in Table S1).

### 2.3. Excision and Transfer of ICEs and IMEs in S. suis

Two strains carrying putative ICEs—NSUI084 and NSUI086—were selected for the experimental part of the work. Strain NSUI084 carries two ICEs: an ICE of the Tn*1549* family (ICE_*SsuNSUI084_rplL_1*) in accretion with an ICE of the Tn*5252* family (ICE_*SsuNSUI084_rplL_2* integrated into the *rplL* gene as shown in Figure 7). This latter hosts an IME integrated into its SNF2 gene. Strain NSUI086 carries an ICE of the Tn*5252* family that hosts an IME integrated into the PPI gene of the ICE (ICE_*SsuNSUI086_rplL* shown in Figure 7).

**Figure 9:**
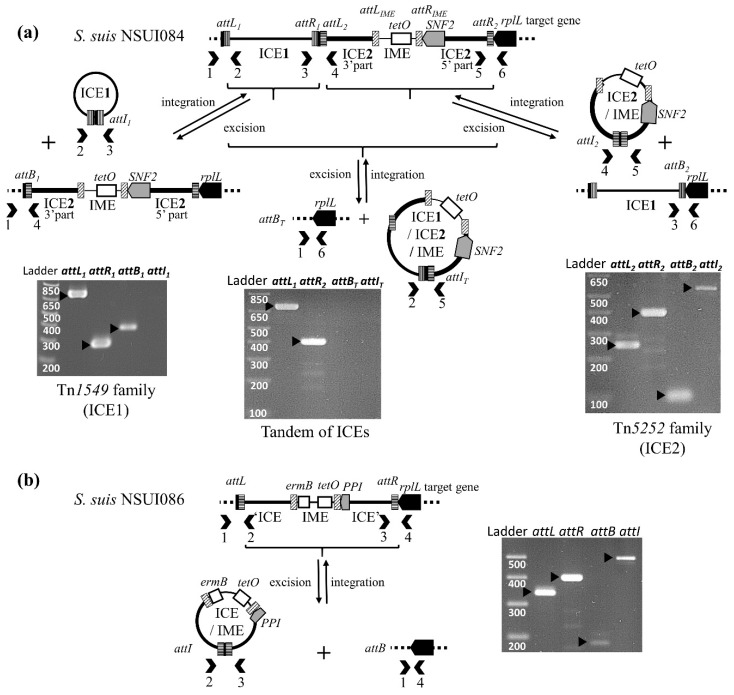
PCR detection of integrated and excised forms of: (**a**) ICEs of strain NSUI084: whole tandem structure or Tn*1549* (ICE1) and Tn*5252* (ICE2) separate elements; (**b**) ICE_*SsuNSUI086_rplL*. The sizes of the PCR fragments obtained for the amplification of controls *attL* (left site of the integrated element) and *attR* (right site of the integrated element), *attB* (empty site) and *attI* (circular form) were confirmed by parallel migration of a DNA ladder. The primers pairs used for these amplifications are listed in Table S2.

PCR experiments were carried out in order to evaluate the excision ability of the ICEs and the hosted IMEs. For strain NSUI084, PCR results indicated that elements of the Tn*1549* and Tn*5252* families are able to excise as separate elements but not as a Tn*1549*-Tn*5252* tandem. By contrast, IME_*SsuNSUI084_SNF2* does not appear to excise, at least in the laboratory conditions tested (Figure 9). For strain NSUI086, the Tn*5252*-related element was able to excise but not IME_*SsuNSUI086_PPI* (Figure 9).

Whereas IME_*NSUI084_SNF2* fulfills all the requirements for an active IME, a thorough sequence re-analysis of IME_*SsuNSUI086_PPI* revealed that it is likely a defective IME (and thus should not be called IME). Indeed, the *erm*(B) gene and two other CDSs are carried by a genetic element—a 1.7 kb-*erm*(B) element—that is integrated into the serine integrase gene of the IME, leading to a truncated gene. This *erm*(B) element is related to the one described in Tn*6002* ICE [27]. PCR experiments—by both PCR and nested PCR—did not show excision of the *erm*(B) element (data not shown).

Many defective elements related to IME_*NSUI084_SNF2* and IME_*SsuNSUI086_PPI* or signatures of the integration of these IMEs—truncated SNF2 or PPI genes—were detected in the genomes (Figure 7 and Table S1).

**Figure 10:**
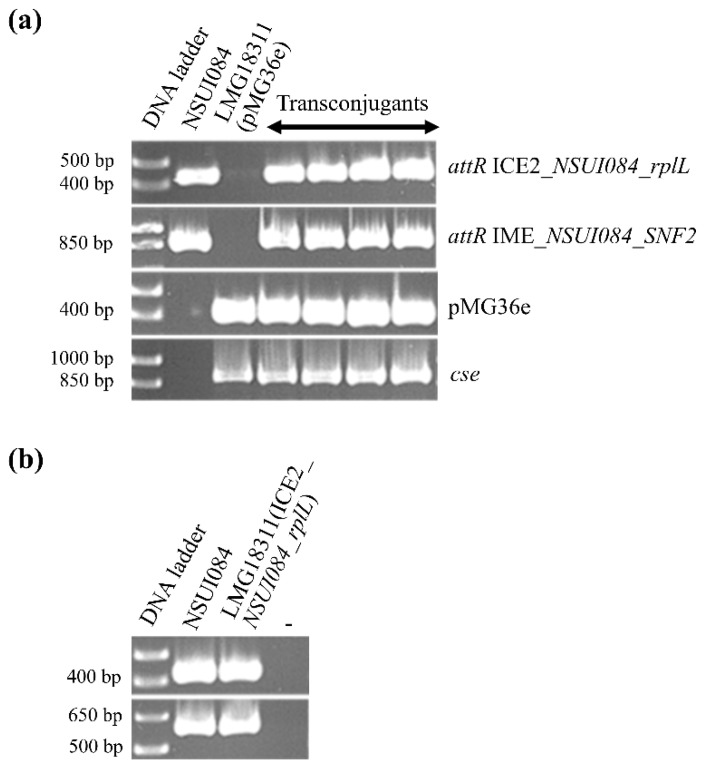
Characterization of transconjugants of *S. thermophilus* LMG18311 (pMG36e) carrying ICE_*SsuNSUI084_rplL_2* obtained after interspecies mating with *S. suis* NSUI084. (**a**) PCR experiments were done to amplify the right attachment site of ICE_*SsuNSUI084_rplL_2* (*attR*_Tn*5252*_84) and of IME_SNF2 hosted by this ICE (*attR*_IME_84), the *erm*(B) gene carried by pMG36e (pMG36e) and a gene-specific of *S. thermophilus* (*cse*); (**b**) excision tests on one of these transconjugants (LMG18311 Tn*5252* by comparison with the control donor strain NSUI084) before using it in retransfer experiments, *attR* on the upper panel and *attI* detection on the lower panel (-: negative control of PCR mix without DNA).

Conjugation experiments were carried out in order to evaluate the conjugative transfer of Tn*5252*-related ICEs from strains NSUI084 and NSUI086 (ICE_*SsuNSUI084_rplL* and ICE_*SsuNSUI086_rplL* respectively). Whatever the donor, no transconjugant was obtained using *S. suis* NSUI029 strain as a recipient (frequency below the detection limit of 10^−7^ per donor cells). By contrast, a conjugative transfer of ICE_*SsuNSUI084_rplL_2*—the Tn*5252*-related ICE part of the composite element—was obtained using *S. thermophilus* LMG18311 (pMG36e) as a recipient (at a high frequency of 2 × 10^−3^ ± 1.10^−3^ per donor cells). This ICE was still able to excise from the *rplL* site in the transconjugants (Figure 10), but as in the initial donor strain, IME_*NSUI084_SNF2* hosted by the ICE was still unable to excise (data not shown). One of these transconjugants was used as a donor in mating experiments after plasmid curing with *S. thermophilus* LMG18311 (pMG36e) as a recipient. Transconjugants were obtained indicating that the ICE is able to retransfer (with a similar frequency) (Figure S2).

The viability of recipient cells of *S. suis* NSUI029 and *S. thermophilus* (LMG18311 (pMG36e) or JIM8232 (pMG36e)) was affected after contact with the NSUI086 *S. suis* donor strain. Further testing revealed that this strain produces a bacteriocin that was toxic for these recipient strains (Figure S3). Strains from two species that appeared resistant to this bacteriocin (*Enterococcus faecalis* JH2-2 (pMG36c) and *Streptococcus salivarius* JIM8777 (pMG36c), Figure S3) were thus tested as recipient cells in following mating assays. However, conjugative transfer of ICE_*SsuNSUI086_rplL* was not observed even when using these strains (indicating a frequency below the detection limit of 10^−7^ per donor cells).

### 2.4. Suicin Synthesis Clusters Carried by ICEs in S. suis

Observations made during conjugation experiments with strain NSUI086 prompted us to search for the presence of bacteriocin synthesis clusters in our set of 214 genomes of *S. suis*. Three suicin synthesis clusters have been described in *S. suis*: suicin 65, 90–1330, and 3908 synthesis clusters [28,29,30]. These clusters have already been described in the ST25 and ST28 strains analyzed in this study [31].

Our sequence analysis indicated that suicin 65 clusters are located on ICEs of the Tn*5252* family integrated into the *rplL* gene (as for example in ICE_*SsuNSUI086_rplL*, Figure 7). Fifteen strains belonging to various serotypes carried a complete suicin 65 cluster of genes.

A full suicin 90-1330 cluster of genes was found on an ICE in four strains (as in the published ICE ICE_*SsuCZ130302-rplL* [16]).

A partial suicin 3908 cluster of genes was detected on an ICE in one strain of our collection (as in ICE_*Ssu05ZYH33_rplL* [32]) (Table S1, Figure 7).

## 3. Discussion

This large-scale genome analysis reveals the high diversity of putative MGEs transferring by conjugation (ICEs and IMEs) in *S. suis*.

In addition to the five families of ICEs already reported in *S. suis* (Tn*5252*, Tn*1549*, Tn*GBS2, Tn916,* and *vanG)* [13,15], ICEs of the ICE*St3* and Tn*GBS1* families were also detected in a few strains. These families are frequent in other streptococci [21,33,34] but were not reported in *S. suis* until now. As described in previous studies, Tn*5252*-related ICEs appear as the most prevalent ones. A new chromosomal integration site (ADP ribose pyrophosphatase gene) was detected in this work for this family, leading to a total of six different reported target genes for Tn*5252*-related ICEs. The second most prevalent family of ICEs is Tn*1549*-related elements. As already described by Huang et al. [13], most of them are integrated in tandem with Tn*5252*-related elements. Thus, whereas accretion of ICEs is rarely described in bacteria, Tn*1549*-Tn*5252* tandem accretion appears very frequent in *S. suis*. ICEs of the other families appear less frequent (2–16 elements with full sequence detected in the genomes). It also appears that ICEs of the same family can co-exist in the same strain. This likely contributes to the evolution of the elements by enabling gene or module exchanges. Evidence of such recombination events was obtained by pairwise-comparison of ICEs.

Putative IMEs appear even more widespread than ICEs in *S. suis*. A thorough analysis of these genetic elements indicates a huge diversity of integration specificities (13 different ones). Some of these integration sites (PPI, three sites inside ICEs of the Tn*1549* family and gene encoding a HTH-XRE regulator) are reported here for the first time. This further extends the repertoire of IMEs found in Streptococci [23]. Twenty-two different combinations of integrase-relaxase-(coupling protein) were observed. This indicates a high dynamics of evolution and plasticity of IMEs as suggested previously [22]. As for ICEs, tandem accretion of IMEs also exists, enabling gene exchange and increasing the evolution potential of these elements. As observed in a previous study on complete genomes of streptococci [22], we did not find IMEs carrying canonical relaxases of the Mob_F_ or Mob_H_ families but the four other canonical families (MOB_P_, MOB_Q_, MOB_V,_ and MOB_C_) were detected. More importantly, half of the IMEs harbor a putative non-canonical relaxase, i.e., related to RCR initiators (MOB_T_, PF01719, PF01719-helicase, PHA00330 or PF02407) and in most cases these IMEs also encode a coupling protein (always from the TcpA family). As proposed previously [23], IMEs encoding relaxases related to RCR initiators can likely only hijack conjugative elements that encode TcpA. The elements that encode a TcpA coupling protein could probably replace the CP from the T4SS of the helper conjugative element by their own CP to promote their transfer (likely at the expense of the helper element).

In this work, genetic elements encoding an integrase but for which no relaxase was detected were not categorized as IMEs but rather as GIs. This does not mean that they should be considered as not transferable by conjugation but rather that further evidence of possible mobilization is needed. In particular, GI integrated into *rpsI* could be mobilizable by subverting the relaxase and mating apparatus of a co-resident ICE. Indeed, the transfer of such element—mis-annotated as an ICE even if it does not carry any conjugation gene—was described recently (at a very low frequency: 3.7×10^−9^ per donor) [35]. It should be pointed out that such elements are more difficult to detect in the genomes and are likely overlooked. In addition, elements with an incomplete conjugation module that we called dICEs are likely still able to excise and thus could still transfer to other cells by using the conjugation apparatus of the other resident ICEs (mobilization *in trans*).

Almost 400 antimicrobial resistance genes were detected in the 214 genomes analyzed.

A total of 55 of these AMR genes were found on ICEs. Most of the ICEs carrying AMR genes belong to the Tn*5252* family that has already been largely described as AMR genes vehicle in *S. suis* [12,15]. Some of them carry novel combinations of AMR genes compared to ICEs previously described by Huang et al. [13] and Palmieri et al. [15]. AMR genes were also found on four other families (Tn*1549*, Tn*GBS2*, Tn*916* and *vanG* family). An ICE, related to Tn*GBS2* of *Streptococcus agalactiae* [36], that carries three AMR genes was identified in one strain of serotype 9, which is the most important and prevalent serotype causing disease in pigs in many European countries [5]. To our knowledge, this is the first case of Tn*GBS2* element carrying AMR genes. A thorough analysis of this ICE indicated that the cluster of AMR genes (*ant*(6)-Ia, *aph*(3′)-IIIa*, tet*(L) conferring resistance to streptomycin, kanamycin and tetracycline, respectively) could have been brought by the integration of an IME inside the ICE. Indeed, a gene encoding a serine recombinase was detected as well as a gene encoding a relaxase of the Mob_V_ family. The sequence of this putative IME is partial since the two genes are located on separate contigs of the genome, so it is difficult to conclude. Among the Tn*916*-elements, two ICEs—carrying an *erm*(B) gene and integrated into a tRNAArg gene—show a conjugation module distantly related to the one of Tn*916* and are almost identical to Tn*6194* previously characterized in *Clostridioides difficile* CII7 [24]. Conjugative transfer of the ICE of *C. difficile* was previously demonstrated towards *Enterococcus faecalis*. In *C. difficile*, the ICE was found integrated with various genes (including a tRNAArg gene) whereas, as observed in *S. suis*, it was found only in a tRNAArg gene in *E. faecalis* [24]. ICEs of this family are more promiscuous than the other ICE families and could contribute to AMR dissemination not only to streptococci but also to other Firmicutes. An AMR gene (*tet*(W)) was also found on ICEs of the *vanG* family integrated into the *lysS* gene in 2 strains of *S. suis* belonging to serotype 9. These ICEs are almost identical to ICE_*SsuGZ1_lysS* described previously [15].

More surprisingly, more than half of the detected AMR genes are carried by putative IMEs. It is important to mention that this is the first report of IMEs carrying AMR genes in *S. suis*. Most of the IMEs carrying AMR genes are integrated into SNF2 or PPI. They are also carried by ICEs as previously described, but were not recognized as IMEs by the authors of these works [12,14]. These IMEs are related to the *tet*(O) fragment integrated into ICE*Sp2905* described in *Streptococcus pyogenes* [37]. AMR genes were also found on IME integrated into *rpsI*, tRNALeu, and in a gene encoding an HTH-XRE regulator. Interestingly, the *vanZ* gene found on an IME_*tRNALeu* exhibits 100% of identity with the one encoded by an IME (not identified as an IME by the authors) integrated inside a Tn*1549*-related ICE—itself integrated into a Tn*5252*-related ICE—in *Streptococcus lutetiensis* (ICE*Slu*van) [17,26]. This suggests a capture of this resistance gene by different genetic elements rather than clonal dissemination of MGEs carrying this gene.

Some AMR genes were also detected on prophages integrated into *rumA*. Since this gene is also a hotspot of integration of ICEs, this could lead to the formation of composite elements between ICEs and prophages as described previously [15].

ICEs of *S. suis* do not only carry AMR genes but also bacteriocin synthesis clusters. As described recently for suicin 90–1330 [38] and for ICE_*Ssu05ZYH33_rplL* [32]—suicin 65 clusters also appear located on ICEs of the Tn*5252* family integrated into the *rplL* gene. All the three suicins (suicin 65, 90-1330, and 3908) described in *S. suis* appear bactericidal for highly virulent ST1 strains of *S. suis* [28,29,30]. However, the carriage of the corresponding biosynthesis clusters on ICEs will hamper their suggested use as an alternative to antibiotics [39] since these MGEs will contribute to the dissemination of resistance genes to these suicins.

Experimental work was also done in order to evaluate the mobility of several ICEs (ICEs of the Tn*5252* family hosting an IME, in tandem or not with a Tn*1549* ICE). Excision of the whole Tn*5252*-Tn*1549* tandem was not observed but the two ICEs were able to excise separately. The Tn*5252*-related ICE part of the tandem was successfully transferred to *S. thermophilus*. Thus, accretion of the ICEs does not hamper their mobility and interspecies gene transfer can occur. No excision of the IMEs hosted by the ICEs (IME_*SNF2* and IME_*PPI*) was detected in the tested conditions. For one of them (IME_*NSUI086_PPI*), this is due to the insertion of an *erm*(B) element in the gene of the serine integrase that impairs the production of active full-length integrase. This can be viewed as a matrioshka: *erm*(B) element integrated into IME_*PPI* that is itself integrated into an ICE of the Tn*5252* family. The *erm*(B) element found in this putative IME is related to the *erm*(B) element described in the ICE Tn*6002* [27]. In this ICE, which belongs to the Tn*916* family, the *erm*(B) element is integrated into the 3′ end of the relaxase gene of the ICE and extends the length of the gene (adding 71 amino acids at the C terminal end of the relaxase protein, without any other modification). This does not impair the conjugative transfer of Tn*6002* [27]. The other IME studied in this work (IME_*NSUI084_SNF2*) fulfills all the requirements for an active IME but still, its excision was not detected. This suggests that either this IME is not mobile anymore as a single element but is transferred passively by the ICE that hosts it (whose conjugative transfer to *S. thermophilus* was observed), or that the IME excision occurs only in specific conditions not met in our laboratory experiments. Many defective elements related to these IMEs were detected in the genomes, suggesting that after integration in the ICEs, these elements undergo decay and lose their mobility.

## 4. Materials and Methods

### 4.1. Bacterial Strains, Plasmids, and Culture Conditions

Characteristics of the strains used in this study are indicated in Table 1. Two strains of *S. suis* carrying an ICE of the Tn*5252* family—that itself hosts an IME integrated into the SNF2 gene (strain NSUI084) or PPI gene (strain NSUI086)—were selected as donor strains for the conjugation experiments. Transfer was followed using the AMR genes—*tet*(O) or *tet*(O)/*erm*(B)—carried by the IMEs. Strain NSUI029 is susceptible to erythromycin and tetracycline and does not carry an ICE of the Tn*5252* family at the *rplL* site. NSUI029 (pMG36c) that carries a plasmid conferring chloramphenicol resistance was used as a recipient. Strains of other species were also tested as recipients in mating experiments (*Streptococcus salivarius* JIM8777, *S. thermophilus* LMG18311 and JIM8232, *E. faecalis* JH2-2). *S. suis*, *S. thermophilus* and *E. faecalis* strains were grown in M17 broth supplemented with 0.5% lactose (LM17) at 37 °C. When required, cultures were supplemented with the following antibiotic concentrations: tetracycline, 6 mg/L; erythromycin, 5 mg/L; chloramphenicol, 4 mg/L.

### 4.2. Nucleotide Sequence Accession Numbers

Bacterial genomes analyzed in this work were sequenced previously [11,40,45]. DNA sequencing short-reads (see accession numbers listed in Table S1) were retrieved from the Sequence Read Archive of NCBI (https://www.ncbi.nlm.nih.gov/sra). *De novo* assemblies were carried out with the A5 pipeline [46]. Contigs were annotated with Prokka (v. 1.12, [47]).

### 4.3. Search for ICEs, IMEs and for Resistance Genes in Streptococcal Genomes

The overall workflow of the strategy used to detect, characterize, and delineate ICEs and IMEs in streptococcal genomes was described previously [10,22].

Resistance genes were searched using MegaBlast (using default NCBI parameters and no filtering) against the ARG-annot database [48] (as uploaded on 2017/06/25).

The localization of the resistance genes on chromosomal genetic elements (ICEs, IMEs, prophages or other genomic elements) was thoroughly examined by visual inspection of surrounding genes in the genome and analysis of their putative function by CD-search on NCBI website.

### 4.4. Comparative Analysis of Genetic Elements

Pairwise comparisons of elements were performed with Artemis Comparison Tool provided by the Sanger Centre [49]. Protein clustering was done as described previously [22]. Circos6 was used to show the signature protein associations and the content of MGEs of each strain [50].

### 4.5. Phylogenetic Tree Based on MLST Data

A Fasta file with the concatenated sequences of the *aroA*, *cpn60*, *dpr*, *gki*, *mutS*, *recA* and *thrA* genes—according to the MLST scheme of *S. suis* described by King et al. [51]—was generated. These sequences were used to generate a phylogenetic tree using MEGA 7 and the maximum likelihood method (Tamura-Nei model) [52,53].

### 4.6. Excision Tests

PCR amplifications were done to detect left (*attL*) and right (*attR*) MGEs borders, the *attI* site of the circular form of the MGEs and the empty chromosomal integration site *attB* as described previously [54] (see Table S2 for primers used).

### 4.7. Mating Experiments

Mating experiments were done as described in Dahmane et al. [55]. Selection of transconjugants was made at 37 °C (for intraspecies *S. suis* mating), 39 °C (for interspecies from *S. suis* to *S. thermophilus* mating) or 42 °C (for intraspecies *S. thermophilus* mating) using tetracycline and chloramphenicol containing-LM17 plates. Mating frequency was reported as the number of transconjugants per donor CFU.

Plasmid curing of the transconjugants used as donor cells in retro-transfer experiments was done as described previously [55].

### 4.8. Tests of Production of Bacteriocin

The multilayer method was done as described by Fontaine et al. [56]. Briefly, a fresh culture of donor *S. suis* strains at an OD600 of 1 was diluted 106-fold in 6 mL of prewarmed soft LM17 (0.8% agar) and poured on a solid LM17 plate. After a 24 h-incubation at 37 °C, a second 6 mL soft LM17 layer of 40-fold diluted fresh culture of the recipient strains at an OD600 of 1 was poured on the top. Plates were incubated 12 h at an optimal growth temperature of the recipient strain and the presence of an inhibition zone surrounding the donor colonies was evaluated.

## 5. Conclusions

Taken together, our results indicate high diversity and prevalence of integrated MGEs carrying AMR genes and transmissible by conjugation in *S. suis*.

A large part of them corresponds to ICEs and dICEs belonging to five families of ICEs (Tn*5252*, Tn*916*, Tn*GBS2*, Tn*1549,* and *vanG*). Since most of these five families of ICEs are largely present in other streptococcal species and even in other Firmicutes, they certainly participate in the dissemination of AMR genes along the food chain but also in the environment.

More importantly, more than half of the AMR genes detected in *S. suis* genomes are carried by IMEs. This new finding indicates that this other category of integrative MGEs transferring by conjugation that has been very poorly studied until now likely plays a major role in the dissemination of AMR genes inside the *S. suis* species but also to other species sharing the same ecosystem.

Further studies are needed to evaluate such gene fluxes inside and between ecosystems and the contribution of ICEs and IMEs in these gene transfers bearing in mind a One Health global perspective.

## Figures and Tables

**Table 1:** Strains used in the experimental part of the work.

Strains	Relevant Phenotype or Genotype	Sequence Type	Source or Reference
*S. suis*			
NSUI029	Wild-type (WT) pig isolate devoid of Tn*5252* element at the *rplL* site (but carrying an element of the Tn*1549* family at this site), Tet^S 1^ and Ery^S 2^	ST28	[40]
NSUI029 (pMG36c)	NSUI029 carrying pMG36c, a plasmid conferring chloramphenicol resistance, Cm^R 3^	ST28	This work
NSUI084	WT pig isolate carrying putative ICEs of the Tn*1549* (ICE_*SSuNSUI084_rplL_1*) and the Tn*5252* (ICE_*SsuNSUI084_rplL_2*) families. The latter hosts an IME (IME_*SsuNSUI084_SNF2*) that carries a *tet*(O) gene, Tet^R 1^	ST28	[40]
NSUI086	WT pig isolate carrying a putative ICE of the Tn*5252* family (ICE_*SsuNSUI086_rplL*) which hosts an IME (IME_*SsuNSUI086_PPI*) that carries a *tet*(O) and an *erm*(B) gene, Tet^R 1^ and Ery^R 2^	ST28	[40]
*S. salivarius*			
JIM8777	WT commensal strain with an empty *rplL* site		[41]
JIM8777 (pMG36e)	JIM8777 carrying pMG36e, a plasmid conferring erythromycin resistance, Ery^R 2^		[42]
*S. thermophilus*			
LMG18311	WT strain with empty *rplL* site		BCCM/LMG
LMG18311 (pMG36e)	LMG18311 carrying pMG36e, a plasmid conferring erythromycin resistance, Ery^R 2^		[43]
JIM8232	WT strain carrying a putative ICE of the Tn*5252* family in the *rplL* gene and IMEs in the *rpsI*, tRNALys and tRNALeu genes.		[44]
JIM8232 (pMG36e)	JIM8232 carrying pMG36e, a plasmid conferring erythromycin resistance, Ery^R 2^		This work
*Enterococcus faecalis*			[43]
JH2-2 (pMG36e)	JH2-2 carrying the plasmid pMG36e conferring erythromycin resistance, Ery^R 2^		

^1^ Tet^S or R^, susceptibility or resistance to tetracycline; ^2^ Ery^S or R^, susceptibility or resistance to erythromycin; ^3^ Cm^R^, resistance to chloramphenicol.

## Supplementary Materials

The following are available online at https://www.mdpi.com/2076-0817/9/1/22/s1, Figure S1: Co-occurrence of relaxases, CPs and integrases among IMEs. Figure S2: Characterization of the transconjugants obtained after intraspecies transfer of ICE_*SsuNSUI084_rplL_2* from *S. thermophilus* LMG18311(ICE_*SsuNSUI084_rplL_2*) to *S. thermophilus* LMG18311(pMG36e). Figure S3: Tests of production of bacteriocin by *S. suis* NSUI086 using a lawn of different indicative bacteria. Table S1: MGE genome content. Table S2: Primers used in this study to detect the integration and excision of putative mobile elements.

## References

[1] Feng Y., Zhang H., Wu Z., Wang S., Cao M., Hu D., Wang C. (2014). *Streptococcus suis* infection: An emerging/reemerging challenge of bacterial infectious diseases?. Virulence.

[2] Huong V.T., Ha N., Huy N.T., Horby P., Nghia H.D., Thiem V.D., Zhu X., Hoa N.T., Hien T.T., Zamora J. (2014). Epidemiology, clinical manifestations, and outcomes of *Streptococcus suis* infection in humans. Emerg. Infect. Dis..

[3] Eisenberg T., Hudemann C., Hossain H.M., Hewer A., Tello K., Bandorski D., Rohde M., Valentin-Weigand P., Baums C.G. (2015). Characterization of five zoonotic *Streptococcus suis* strains from Germany, including one isolate from a recent fatal case of streptococcal toxic shock-like syndrome in a hunter. J. Clin. Microbiol..

[4] Gomez-Torres J., Nimir A., Cluett J., Aggarwal A., Elsayed S., Soares D., Teatero S., Chen Y., Gottschalk M., Fittipaldi N. (2017). Human case of *Streptococcus suis* disease, Ontario, Canada. Emerg. Infect. Dis..

[5] Goyette-Desjardins G., Auger J.P., Xu J., Segura M., Gottschalk M. (2014). *Streptococcus suis*, an important pig pathogen and emerging zoonotic agent-an update on the worldwide distribution based on serotyping and sequence typing. Emerg. Microbes. Infect..

[6] Kerdsin A., Hatrongjit R., Gottschalk M., Takeuchi D., Hamada S., Akeda Y., Oishi K. (2017). Emergence of *Streptococcus suis* serotype 9 infection in humans. J. Microbiol. Immunol. Infect..

[7] European Centre for Disease Prevention and Control (2018). Surveillance of Antimicrobial Resistance in Europe 2017. Annual Report of the European Antimicrobial Resistance Surveillance Network (EARS-Net).

[8] HHS (2017). The National Antimicrobial Resistance Monitoring System: NARMS 2015 Integrated Report.

[9] Varela N.P., Gadbois P., Thibault C., Gottschalk M., Dick P., Wilson J. (2013). Antimicrobial resistance and prudent drug use for *Streptococcus suis*. Anim. Health. Res. Rev..

[10] Ambroset C., Coluzzi C., Guédon G., Devignes M.D., Loux V., Lacroix T., Payot S., Leblond-Bourget N. (2016). New insights into the classification and integration specificity of *Streptococcus* integrative conjugative elements through extensive genome exploration. Front. Microbiol..

[11] Athey T.B., Teatero S., Takamatsu D., Wasserscheid J., Dewar K., Gottschalk M., Fittipaldi N. (2016). Population structure and antimicrobial resistance profiles of *Streptococcus suis* serotype 2 sequence type 25 strains. PLoS ONE.

[12] Huang J., Liang Y., Guo D., Shang K., Ge L., Kashif J., Wang L. (2016). Comparative genomic analysis of the ICES*a2603* family ICEs and spread of *erm*(B)- and *tet*(O)-carrying transferable 89K-subtype ICEs in swine and bovine isolates in china. Front. Microbiol..

[13] Huang J., Ma J., Shang K., Hu X., Liang Y., Li D., Wu Z., Dai L., Chen L., Wang L. (2016). Evolution and diversity of the antimicrobial resistance associated mobilome in *Streptococcus suis*: A probable mobile genetic elements reservoir for other streptococci. Front. Cell. Infect. Microbiol..

[14] Huang K., Song Y., Zhang Q., Zhang A., Jin M. (2016). Characterisation of a novel integrative and conjugative element ICE*SsD9* carrying *erm*(B) and *tet*(O) resistance determinants in *Streptococcus suis*, and the distribution of ICE*SsD9*-like elements in clinical isolates. J. Glob. Antimicrob. Resist..

[15] Palmieri C., Varaldo P.E., Facinelli B. (2011). *Streptococcus suis*, an emerging drug-resistant animal and human pathogen. Front. Microbiol..

[16] Pan Z., Liu J., Zhang Y., Chen S., Ma J., Dong W., Wu Z., Yao H. (2019). A novel integrative conjugative element mediates transfer of multi-drug resistance between *Streptococcus suis* strains of different serotypes. Vet. Microbiol..

[17] Bellanger X., Payot S., Leblond-Bourget N., Guédon G. (2014). Conjugative and mobilizable genomic islands in bacteria: Evolution and diversity. FEMS Microbiol. Rev..

[18] Grohmann E., Christie P.J., Waksman G., Backert S. (2018). Type IV secretion in gram-negative and gram-positive bacteria. Mol. Microbiol..

[19] Holden M.T., Hauser H., Sanders M., Ngo T.H., Cherevach I., Cronin A., Goodhead I., Mungall K., Quail M.A., Price C. (2009). Rapid evolution of virulence and drug resistance in the emerging zoonotic pathogen *Streptococcus suis*. PLoS ONE.

[20] Zheng H., Du P., Qiu X., Kerdsin A., Roy D., Bai X., Xu J., Vela A.I., Gottschalk M. (2018). Genomic comparisons of *Streptococcus suis* serotype 9 strains recovered from diseased pigs in Spain and Canada. Vet. Res..

[21] Burrus V., Pavlovic G., Decaris B., Guédon G. (2002). The ICES*t1* element of *Streptococcus thermophilus* belongs to a large family of integrative and conjugative elements that exchange modules and change their specificity of integration. Plasmid.

[22] Coluzzi C., Guédon G., Devignes M.D., Ambroset C., Loux V., Lacroix T., Payot S., Leblond-Bourget N. (2017). A glimpse into the world of integrative and mobilizable elements in streptococci reveals an unexpected diversity and novel families of mobilization proteins. Front. Microbiol..

[23] Guédon G., Libante V., Coluzzi C., Payot S., Leblond-Bourget N. (2017). The obscure world of integrative and mobilizable elements, highly widespread elements that pirate bacterial conjugative systems. Genes.

[24] Wasels F., Monot M., Spigaglia P., Barbanti F., Ma L., Bouchier C., Dupuy B., Mastrantonio P. (2014). Inter- and intraspecies transfer of a *Clostridium difficile* conjugative transposon conferring resistance to MLS_B_. Microb. Drug Resist..

[25] Lai L., Dai J., Tang H., Zhang S., Wu C., Qiu W., Lu C., Yao H., Fan H., Wu Z. (2017). *Streptococcus suis* serotype 9 strain GZ0565 contains a type VII secretion system putative substrate EsxA that contributes to bacterial virulence and a *vanZ*-like gene that confers resistance to teicoplanin and dalbavancin in *Streptococcus agalactiae*. Vet. Microbiol..

[26] Bjorkeng E.K., Hjerde E., Pedersen T., Sundsfjord A., Hegstad K. (2013). ICES*lu*van, a 94-kilobase mosaic integrative conjugative element conferring interspecies transfer of VanB-type glycopeptide resistance, a novel bacitracin resistance locus, and a toxin-antitoxin stabilization system. J. Bacteriol..

[27] Warburton P.J., Palmer R.M., Munson M.A., Wade W.G. (2007). Demonstration of in vivo transfer of doxycycline resistance mediated by a novel transposon. J. Antimicrob. Chemother..

[28] LeBel G., Vaillancourt K., Frenette M., Gottschalk M., Grenier D. (2014). Suicin 90-1330 from a nonvirulent strain of *Streptococcus suis*: A nisin-related lantibiotic active on gram-positive swine pathogens. Appl. Environ. Microbiol..

[29] Vaillancourt K., LeBel G., Frenette M., Fittipaldi N., Gottschalk M., Grenier D. (2015). Purification and characterization of suicin 65, a novel class I type B lantibiotic produced by *Streptococcus suis*. PLoS ONE.

[30] Vaillancourt K., LeBel G., Frenette M., Gottschalk M., Grenier D. (2015). Suicin 3908, a new lantibiotic produced by a strain of *Streptococcus suis* serotype 2 isolated from a healthy carrier pig. PLoS ONE.

[31] Athey T.B., Vaillancourt K., Frenette M., Fittipaldi N., Gottschalk M., Grenier D. (2016). Distribution of suicin gene clusters in *Streptococcus suis* serotype 2 belonging to sequence types 25 and 28. BioMed Res. Int..

[32] Li M., Shen X., Yan J., Han H., Zheng B., Liu D., Cheng H., Zhao Y., Rao X., Wang C. (2011). GI-type T4SS-mediated horizontal transfer of the 89K pathogenicity island in epidemic *Streptococcus suis* serotype 2. Mol. Microbiol..

[33] Brochet M., Couve E., Glaser P., Guédon G., Payot S. (2008). Integrative conjugative elements and related elements are major contributors to the genome diversity of *Streptococcus agalactiae*. J. Bacteriol..

[34] Puymège A., Bertin S., Guédon G., Payot S. (2015). Analysis of *Streptococcus agalactiae* pan-genome for prevalence, diversity and functionality of integrative and conjugative or mobilizable elements integrated in the tRNA(Lys CTT) gene. Mol. Genet. Genom..

[35] Huang K., Zhang Q., Song Y., Zhang Z., Zhang A., Xiao J., Jin M. (2016). Characterization of spectinomycin resistance in *Streptococcus suis* leads to two novel insights into drug resistance formation and dissemination mechanism. Antimicrob. Agents Chemother..

[36] Guérillot R., Da Cunha V., Sauvage E., Bouchier C., Glaser P. (2013). Modular evolution of Tn*GBS*s, a new family of integrative and conjugative elements associating insertion sequence transposition, plasmid replication, and conjugation for their spreading. J. Bacteriol..

[37] Giovanetti E., Brenciani A., Tiberi E., Bacciaglia A., Varaldo P.E. (2012). ICES*p2905*, the *erm*(TR)-*tet*(O) element of *Streptococcus pyogenes*, is formed by two independent integrative and conjugative elements. Antimicrob. Agents Chemother..

[38] Sun Y., Veseli I.A., Vaillancourt K., Frenette M., Grenier D., Pombert J.F. (2019). The bacteriocin from the prophylactic candidate *Streptococcus suis* 90-1330 is widely distributed across *S. suis* isolates and appears encoded in an integrative and conjugative element. PLoS ONE.

[39] Cotter P.D., Ross R.P., Hill C. (2013). Bacteriocins—A viable alternative to antibiotics?. Nat. Rev. Microbiol..

[40] Athey T.B., Auger J.P., Teatero S., Dumesnil A., Takamatsu D., Wasserscheid J., Dewar K., Gottschalk M., Fittipaldi N. (2015). Complex population structure and virulence differences among serotype 2 *Streptococcus suis* strains belonging to sequence type 28. PLoS ONE.

[41] Guédon E., Delorme C., Pons N., Cruaud C., Loux V., Couloux A., Gautier C., Sanchez N., Layec S., Galleron N. (2011). Complete genome sequence of the commensal *Streptococcus salivarius* strain JIM8777. J. Bacteriol..

[42] Dahmane N., Libante V., Charron-Bourgoin F., Guédon E., Guédon G., Leblond-Bourget N., Payot S. (2017). Diversity of integrative and conjugative elements of *Streptococcus salivarius* and their intra-and interspecies transfer. Appl. Environ. Microbiol..

[43] Bellanger X., Roberts A.P., Morel C., Choulet F., Pavlovic G., Mullany P., Decaris B., Guédon G. (2009). Conjugative transfer of the integrative conjugative elements ICES*t1* and ICES*t3* from *Streptococcus thermophilus*. J. Bacteriol..

[44] Delorme C., Bartholini C., Luraschi M., Pons N., Loux V., Almeida M., Guédon E., Gibrat J.F., Renault P. (2011). Complete genome sequence of the pigmented *Streptococcus thermophilus* strain JIM8232. J. Bacteriol..

[45] Athey T.B., Teatero S., Lacouture S., Takamatsu D., Gottschalk M., Fittipaldi N. (2016). Determining *Streptococcus suis* serotype from short-read whole-genome sequencing data. BMC Microbiol..

[46] Tritt A., Eisen J.A., Facciotti M.T., Darling A.E. (2012). An integrated pipeline for *de novo* assembly of microbial genomes. PLoS ONE.

[47] Seemann T. (2014). Prokka: Rapid prokaryotic genome annotation. Bioinformatics.

[48] Gupta S.K., Padmanabhan B.R., Diene S.M., Lopez-Rojas R., Kempf M., Landraud L., Rolain J.M. (2014). Arg-annot, a new bioinformatic tool to discover antibiotic resistance genes in bacterial genomes. Antimicrob. Agents Chemother..

[49] Carver T., Berriman M., Tivey A., Patel C., Bohme U., Barrell B.G., Parkhill J., Rajandream M.A. (2008). Artemis and act: Viewing, annotating and comparing sequences stored in a relational database. Bioinformatics.

[50] Krzywinski M., Schein J., Birol I., Connors J., Gascoyne R., Horsman D., Jones S.J., Marra M.A. (2009). Circos: An information aesthetic for comparative genomics. Genome. Res..

[51] King S.J., Leigh J.A., Heath P.J., Luque I., Tarradas C., Dowson C.G., Whatmore A.M. (2002). Development of a multilocus sequence typing scheme for the pig pathogen *Streptococcus suis*: Identification of virulent clones and potential capsular serotype exchange. J. Clin. Microbiol..

[52] Kumar S., Stecher G., Tamura K. (2016). Mega7: Molecular evolutionary genetics analysis version 7.0 for bigger datasets. Mol. Biol. Evol..

[53] Tamura K., Nei M. (1993). Estimation of the number of nucleotide substitutions in the control region of mitochondrial DNA in humans and chimpanzees. Mol. Biol. Evol..

[54] Carraro N., Libante V., Morel C., Charron-Bourgoin F., Leblond P., Guédon G. (2016). Plasmid-like replication of a minimal streptococcal integrative and conjugative element. Microbiology.

[55] Dahmane N., Robert E., Deschamps J., Meylheuc T., Delorme C., Briandet R., Leblond-Bourget N., Guédon E., Payot S. (2018). Impact of cell surface molecules on conjugative transfer of the integrative and conjugative element ICES*t3* of *Streptococcus thermophilus*. Appl. Environ. Microbiol..

[56] Fontaine L., Boutry C., Guédon E., Guillot A., Ibrahim M., Grossiord B., Hols P. (2007). Quorum-sensing regulation of the production of Blp bacteriocins in *Streptococcus thermophilus*. J. Bacteriol..

