# Supplementary material for: Chromosomal Conjugative and Mobilizable Elements in Streptococcus suis: Major Actors in the Spreading of Antimicrobial Resistance and Bacteriocin Synthesis Genes
Source: Pathogens. 2019 Dec 25;9(1):22. doi: 10.3390/pathogens9010022 (PMC7168690; doi:10.3390/pathogens9010022)
Supplement: Supplementary file 1 [file pathogens-09-00022-s001.zip › supplementary files/Table S2 primers used.docx]

**TABLE S2.** Primers used in this study to detect integration and excision of putative mobile elements.

| Element | Primer name | Sequence (5’-3’) | PCR fragment (bp) | Reference |
| --- | --- | --- | --- | --- |
| ICE1_*NSUI084_rplL* (Tn*1549* family) |  |  |  |  |
| *attB* | ICENemrplL4 | CATGCTGATAAATCAACAACT | 351 | 1 |
|  | CDS_603_NSUI084_Fwd | TTCATGTCTTTCTCCTAATC |  | This study |
| *attI* | NearDR_NSUI084_Fwd | GCCTTATTACTTGGTTTCT | 619 | This study |
|  | CDS_604_NSUI084_Rev | AACACTTCATGTCCAATC |  | This study |
| *attL* | CDS_604_NSUI084_Rev | AACACTTCATGTCCAATC | 705 | This study |
|  | CDS_603_NSUI084_Fwd | TTCATGTCTTTCTCCTAATC |  | This study |
| *attR* | NearDR_NSUI084_Fwd | GCCTTATTACTTGGTTTCT | 249 | This study |
|  | ICENemrplL4 | CATGCTGATAAATCAACAACT |  | 1 |
| ICE2_*NSUI084_rplL* (Tn*5252* family) |  |  |  |  |
| *attB* | RplLF | CTGAAGAAATCAAAGCTAAA | 104 | This study |
|  | NearDR_NSUI084_Fwd | GCCTTATTACTTGGTTTCT |  | This study |
| *attI* | ICENemrplL4 | CATGCTGATAAATCAACAACT | 535 | 1 |
|  | CDS_701_NSUI084_Fwd | ATGTCGCTTTCTCAATATC |  | This study |
| *attL* | ICENemrplL4 | CATGCTGATAAATCAACAACT | 249 | 1 |
|  | NearDR_NSUI084_Fwd | GCCTTATTACTTGGTTTCT |  | This study |
| *attR* | RplLF | CTGAAGAAATCAAAGCTAAA | 400 | This study |
|  | CDS_701_NSUI084_Fwd | ATGTCGCTTTCTCAATATC |  | This study |
| ICE1-ICE2 tandem in NSUI084 |  |  |  |  |
| *attB* | RplLF | CTGAAGAAATCAAAGCTAAA | 191 | This study |
|  | CDS_603_NSUI084_Fwd | TTCATGTCTTTCTCCTAATC |  | This study |
| *attI* | CDS_604_NSUI084_Rev | AACACTTCATGTCCAATC | 754 | This study |
|  | CDS_701_NSUI084_Fwd | ATGTCGCTTTCTCAATATC |  | This study |
| *attL* | CDS_604_NSUI084_Rev | AACACTTCATGTCCAATC | 705 | This study |
|  | CDS_603_NSUI084_Fwd | TTCATGTCTTTCTCCTAATC |  | This study |
| *attR* | RplLF | CTGAAGAAATCAAAGCTAAA | 400 | This study |
|  | CDS_701_NSUI084_Fwd | ATGTCGCTTTCTCAATATC |  | This study |
| IME_*NSUI084_SNF2* |  |  |  |  |
| *attB -ext-nestPCR* | 84NestRIMER | CCTGAACTCATGCGGATTTA | 1343 | This study |
|  | 84NestLIMEF | TGGTCGTCTAAGATATGG |  | This study |
| *attB -inner-nestPCR* | SNF2_NSUI084_Rev | ATTCTCATGGCTTCTACT | 632 | This study |
|  | SNF2_NSUI084_Fwd | GTTCTTACTTCTTTGGTTTC |  | This study |
| *attI-ext-nestPCR* | 86attINestF | CTCCTATCACTGGGTAAA | 1744 | This study |
|  | 84NestLIMER | GAAGAATGGGAAGTGATAC |  | This study |
| *attI-inner-nestPCR* | attI_IME86_rev | TGCCTATTGCCTGTTCTTC | 452 | This study |
|  | SerRec_NSUI084_Rev | TGTCGGTGAATTACATATC |  | This study |
| ICE_*NSUI086_rplL* (Tn*5252* family) |  |  |  |  |
| *attB* | RplLF | CTGAAGAAATCAAAGCTAAA | 185 | This study |
|  | CDS_603_NSUI084_Fwd | TTCATGTCTTTCTCCTAATC |  | This study |
| *attI* | ICENemrplL4 | CATGCTGATAAATCAACAACT | 492 | 1 |
|  | CDS_701_NSUI084_Fwd | ATGTCGCTTTCTCAATATC |  | This study |
| *attL* | ICENemrplL4 | CATGCTGATAAATCAACAACT | 336 | 1 |
|  | CDS_603_NSUI084_Fwd | TTCATGTCTTTCTCCTAATC |  | This study |
| *attR* | RplLF | CTGAAGAAATCAAAGCTAAA | 400 | This study |
|  | CDS_701_NSUI084_Fwd | ATGTCGCTTTCTCAATATC |  | This study |
| IME_*NSUI086_PPI* |  |  |  |  |
| *attB -ext-nestPCR* | PPIso_NSUI086_Rev | GGAAGCAAATCAGAATAAAG | 622 | This study |
|  | CDS_689_NSUI086_Fwd | CCAGTTTCAAGATAGGATA |  | This study |
| *attB -inner-nestPCR* | Ppisom_86_Fwd | CCGACAAGCTCCTGGTTA | 274 | This study |
|  | attB_IME86_rev | CATGGTCTGGGTGTTCAT |  | This study |
| *attI-ext-nestPCR* | 86_SerRectruncR | GAGGATAAGAAAGCAGATAA | 4509 | This study |
|  | TetO_NSUI084_Fwd | CTATCACTGGGTAAATAACT |  | This study |
| *attI-inner-nestPCR* | 86SerRecNest2R | ttacAGTATGGCAGGAATCA | 3701 | This study |
|  | attI_IME86_rev | TGCCTATTGCCTGTTCTTC |  | This study |
| *ermB* element (NSUI086) |  |  |  |  |
| *ext-nestPCR* | 86_SerRectruncR | GAGGATAAGAAAGCAGATAA | 839 | This study |
|  | 86SerRecNest1F | AATGCAAGGTCGAATATC |  | This study |
| *inner-nestPCR* | 86SerRecNest2R | ttacAGTATGGCAGGAATCA | 295 | This study |
|  | 86_SerRecF | CCAGATATAACCCACAATC |  | This study |

1. Brochet M, Couve E, Glaser P *et al*. Integrative conjugative elements and related elements are major contributors to the genome diversity of *Streptococcus agalactiae*. *J Bacteriol* 2008; **190**:6913-7.
