# Supplementary figures and images for: Chromosomal Conjugative and Mobilizable Elements in Streptococcus suis: Major Actors in the Spreading of Antimicrobial Resistance and Bacteriocin Synthesis Genes
Source: Pathogens. 2019 Dec 25;9(1):22. doi: 10.3390/pathogens9010022 (PMC7168690; doi:10.3390/pathogens9010022)

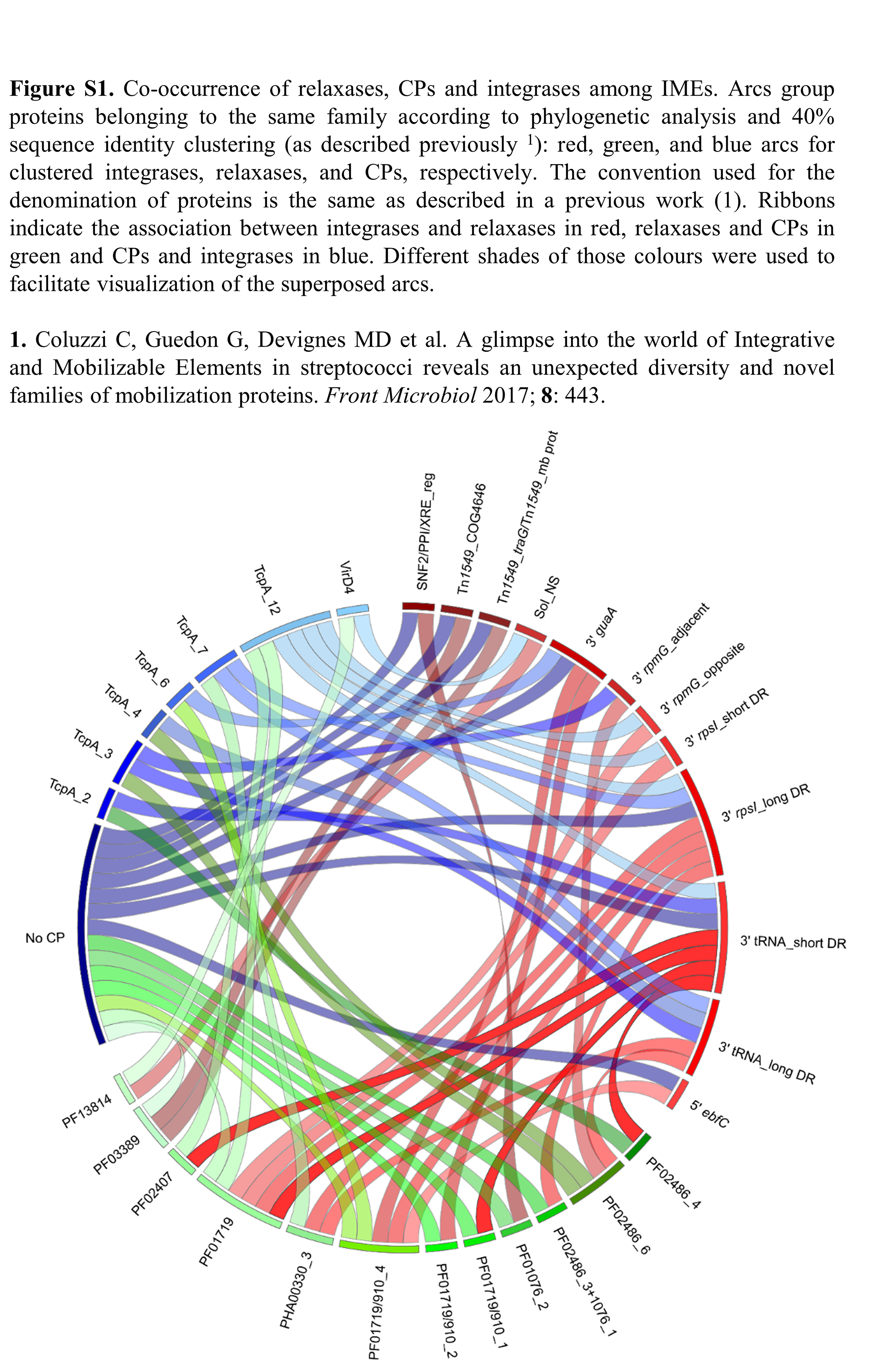

Supplement: Supplementary file 1 [file pathogens-09-00022-s001.zip › supplementary files/Fig S1.tif]

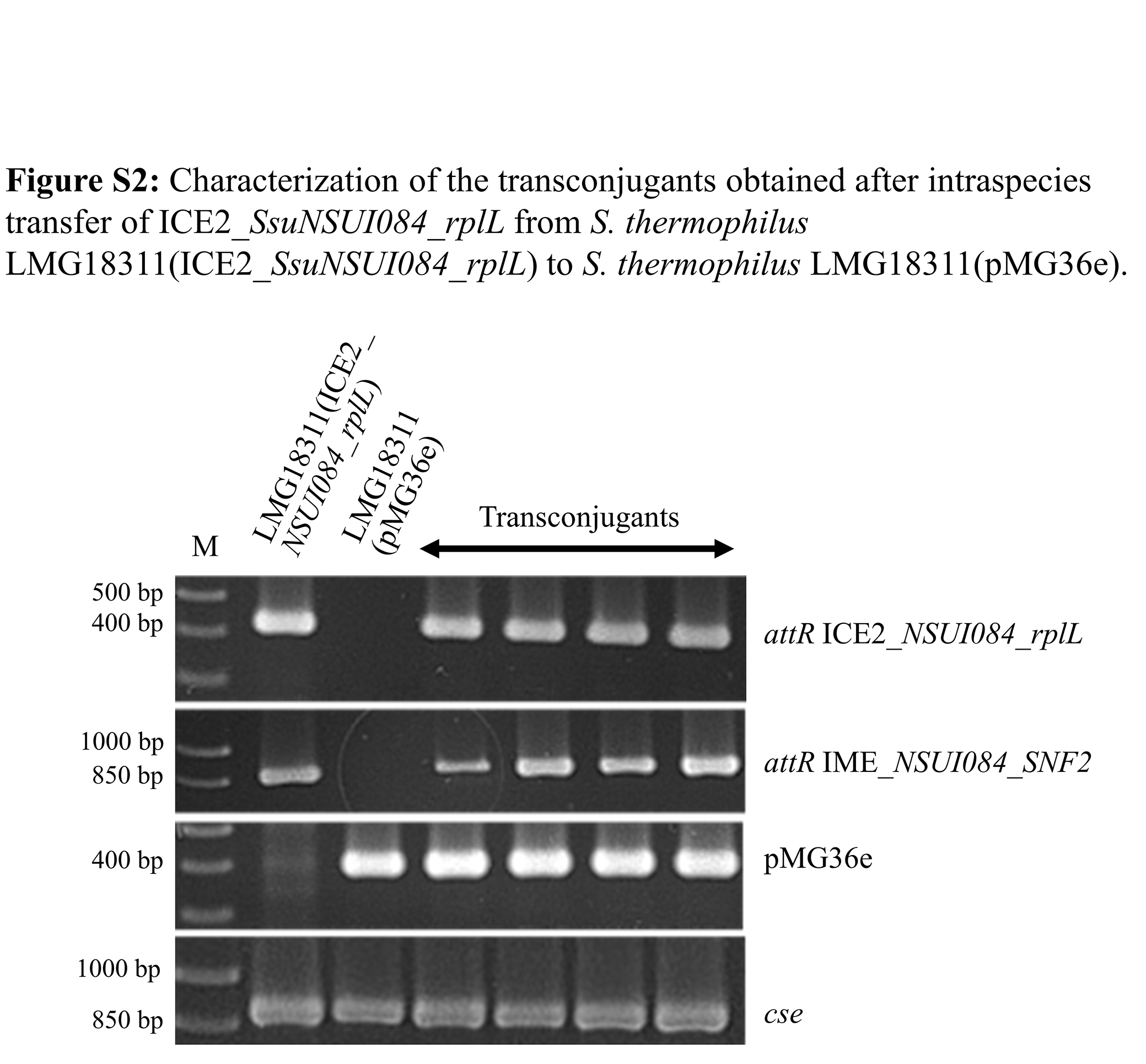

Supplement: Supplementary file 1 [file pathogens-09-00022-s001.zip › supplementary files/Fig S2.tif]

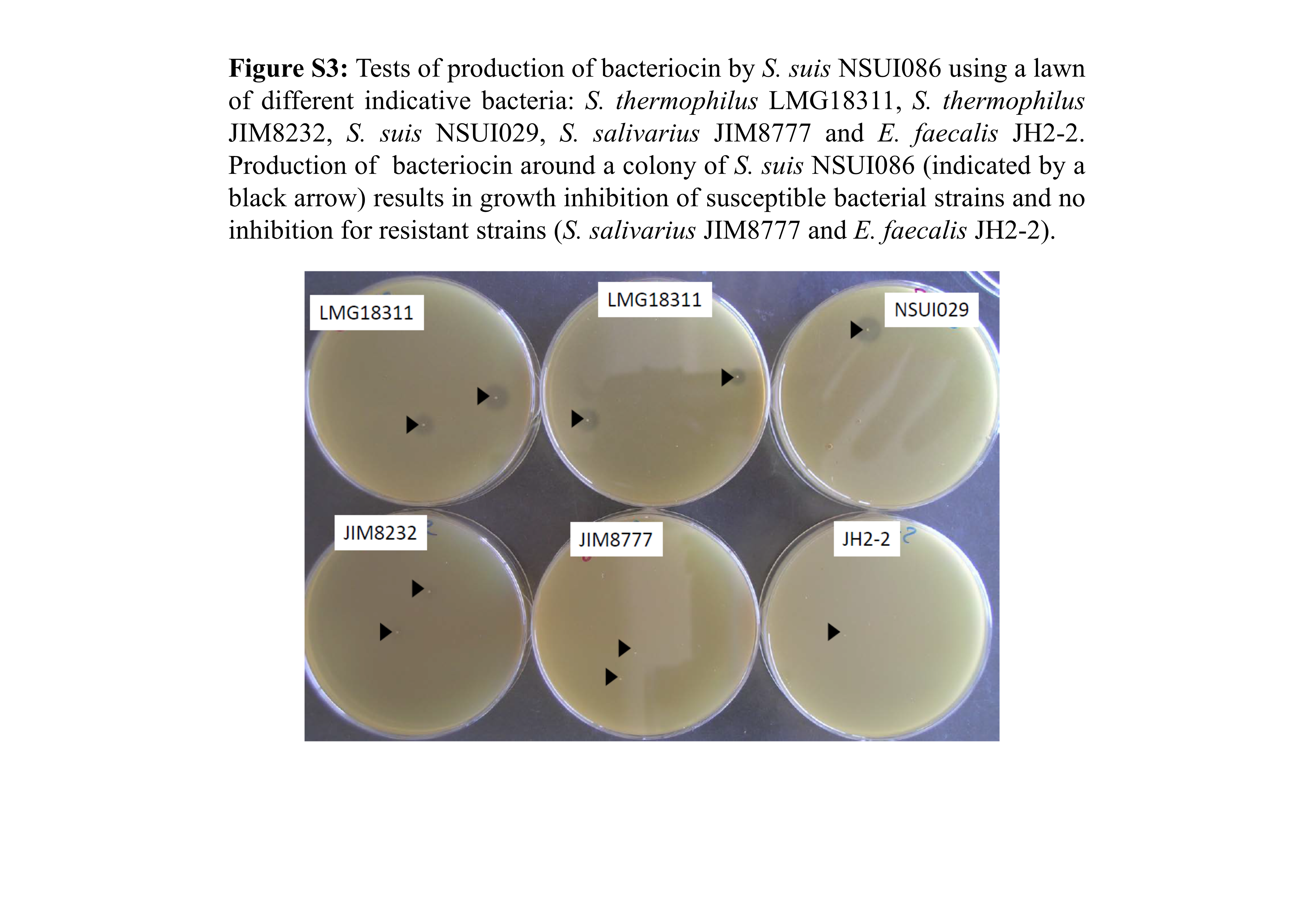

Supplement: Supplementary file 1 [file pathogens-09-00022-s001.zip › supplementary files/Fig S3.tif]
